# *In situ* frequency gating and beam splitting of vacuum- and extreme-ultraviolet pulses

**DOI:** 10.1038/lsa.2016.170

**Published:** 2016-11-18

**Authors:** Rajendran Rajeev, Johannes Hellwagner, Anne Schumacher, Inga Jordan, Martin Huppert, Andres Tehlar, Bhargava Ram Niraghatam, Denitsa Baykusheva, Nan Lin, Aaron von Conta, Hans Jakob Wörner

**Affiliations:** Laboratory of Physical Chemistry, Department of Chemistry and Applied Biosciences, Vladimir-Prelog-Weg 2, ETH Zurich, Zurich 8093, Switzerland

**Keywords:** beam splitting, below-threshold harmonics, coherent extreme-ultraviolet pulses, frequency gating, non-collinear generation

## Abstract

Monochromatization of high-harmonic sources has opened fascinating perspectives regarding time-resolved photoemission from all phases of matter. Such studies have invariably involved the use of spectral filters or spectrally dispersive optical components that are inherently lossy and technically complex. Here we present a new technique for the spectral selection of near-threshold harmonics and their spatial separation from the driving beams without any optical elements. We discover the existence of a narrow phase-matching gate resulting from the combination of the non-collinear generation geometry in an extended medium, atomic resonances and absorption. Our technique offers a filter contrast of up to 10^4^ for the selected harmonics against the adjacent ones and offers multiple temporally synchronized beamlets in a single unified scheme. We demonstrate the selective generation of 133, 80 or 56 nm femtosecond pulses from a 400-nm driver, which is specific to the target gas. These results open new pathways towards phase-sensitive multi-pulse spectroscopy in the vacuum- and extreme-ultraviolet, and frequency-selective output coupling from enhancement cavities.

## Introduction

Ultrashort pulses in the vacuum- and extreme-ultraviolet (VUV/XUV) regimes are central to initiating and following ultrafast dynamics of photo-induced reactions^1, 2, 3^. The use of low photon energies is particularly attractive because it enables background-free photoemission from electronically excited states. Harmonic emission close to the ionization threshold of the gaseous medium has therefore recently attracted considerable interest^4, 5, 6, 7^. An additional motivation for such studies is the rich and incompletely understood mechanism of near-threshold harmonic (NTH) generation^8, 9, 10, 11, 12^. On one hand, the proximity of the corresponding photon energies to the ionization threshold invalidates the traditional treatment of high-harmonic generation based on the strong-field approximation^10, 11, 12^. On the other hand, the field control of the characteristic resonance enhancement near bound atomic states has experimentally reaffirmed the non-perturbative nature of the generation mechanism^5^.

The ideal source for ultrafast time-resolved photoemission studies would provide single harmonic orders separated from both the fundamental and the other harmonic orders. The recently introduced non-collinear geometries^13, 14, 15, 16, 17^ represent an attractive approach to this goal. Unfortunately, the very large number of possible wave-mixing combinations has prevented their applications so far. As a consequence, dispersive gratings have been required in all of those studies, precluding applications in time-resolved photoemission.

In this article, we demonstrate the new concept of a combined all-optical beam splitter and spectral filter in the VUV/XUV reg-imes that are built into the generation medium. Our technique combines the unique phase-matching properties of a non-collinear geometry with the effect of a manifold of resonances and absorption in an extended medium. We demonstrate that the emission can be restricted to a single specific harmonic order with a contrast of up to 10^4^ allowing for both spatial separation and multi-pulse experiments with multiple synchronized beamlets. Working with a 400-nm driving field, we observe the selective emission of harmonic order three from Xe and Kr, five from Ar and seven from Ne.

## Materials and methods

**Figure 1:**
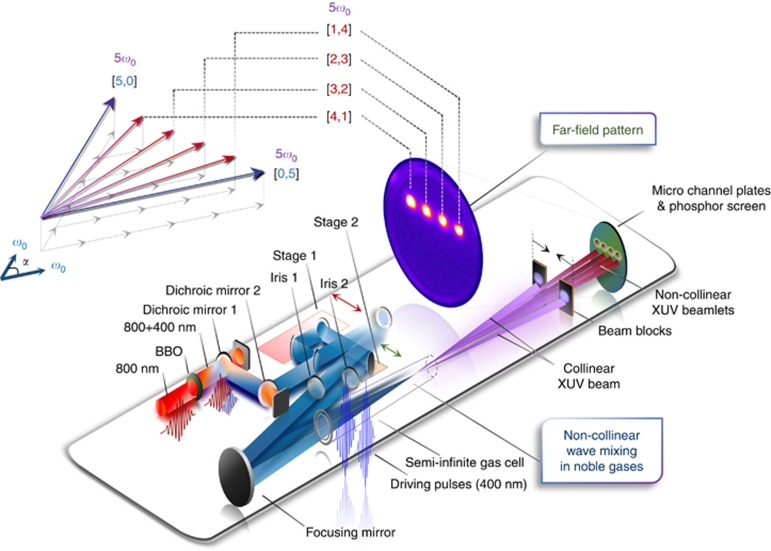
Experimental setup for non-collinear generation of near-threshold harmonics. A 30-fs pulse centered at 800 nm (red) is frequency doubled in a type-I beta-barium-oxide (BBO) crystal. The 400-nm pulse (blue) is isolated from the infrared beam using a pair of dichroic mirrors and is split into two equally intense beams that are subsequently non-collinearly focused into a semi-infinite gas cell using a *f*=50 cm focusing mirror and spatiotemporally matched using translation stage 1 (red double-headed arrows). Irises 1 and 2 control the diameter and intensity of the driving beams, whereas translation stage 2 (green double-headed arrows) determines the lateral beam separation and thus the crossing angle *α* in the medium. Non-collinear wave mixing in rare gases generates multiple beamlets in the emission cone that are recorded using a MCP for position-sensitive detection. The beam blocks in front of the detector are used to prevent the intense collinear beams from saturating the MCP. The vector diagram on the top left illustrates the emission directions (arrows in red) based on momentum conservation for 5*ω*_0_ beamlets in non-collinear wave mixing of two fields of the same photon energy (*ω*_0_) crossing at an angle *α*. The photon contributions [*m*,*n*] from each driving pulse are indicated for the respective beamlets.

The experimental setup is shown in Figure 1. High-harmonic generation from non-collinear wave mixing of linearly polarized driving fields, centered at 400 nm and focused to intensities of 10^12^–10^13^ W cm^−2^, was achieved in a semi-infinite gas cell filled with a rare gas. The far-field emission pattern is visualized directly on a micro-channel plate (MCP) without the use of a grating. Multiple independently pumped compartments in conjunction with a combination of horizontal slits enabled differential pumping downstream for safe high-voltage operation of the MCP. The use of 400-nm fields was motivated by the recent experiments on wavelength-scaling of harmonic intensity in the multiphoton regimes^18, 19^, which showed that the microscopic harmonic intensity *S*_*q*_(*λ*) scaled with wavelength *λ* as *S*_*q*_(*λ*) ∝ *λ*^−4.5^, making a 400-nm driver ~23 times more efficient than 800-nm pulses^19^. In addition, the large inter-harmonic separation of 6.2 eV, characteristic of the 400-nm harmonic spectrum, further simplifies their direct spatial separation and spectral characterization without dispersive optical elements. The emission features in the detection plane were resolved by optimizing the diameter of an iris in each of the two driving beams and by introducing beam blocks in the vacuum chamber to prevent the collinear emission from blinding the detector. The order *q* and photon-number combinations [*m*, *n*] of the XUV beamlets within the emission cone were directly identified from their unique lateral positions in the far field (see arrow diagram in Figure 1) as described below. Here and in what follows, [*m*, *n*] refers to wave-mixing combinations corresponding to *m* photons being contributed by one of the driving fields and *n=q−m* photons by the other.

The intensity of the driving fields were chosen such that NTH dominated the emitted orders. The absence of high harmonics and the presence of only few beamlets thus allowed for their characterization by the unique mapping between lateral position and photon energy *qω*_0_. The emission angle *β*_*q,m*_ for a given harmonic order *q* with respect to the bisector of the crossing angle *α* is given by


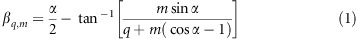


where *m* assumes integer values between 0 and *q*. The collinear beam positions on the MCP, measured by removing the beam blocks and lowering the MCP gain, were used to determine the actual angle *α*. For low values of *q*, the angular variation Δ*β* between beamlets with [*m*, *n*] and [*m*+1, *n*−1] was large enough to be spatially resolved. The use of driver fields with similar intensities was crucial to implement this map.

**Figure 2:**
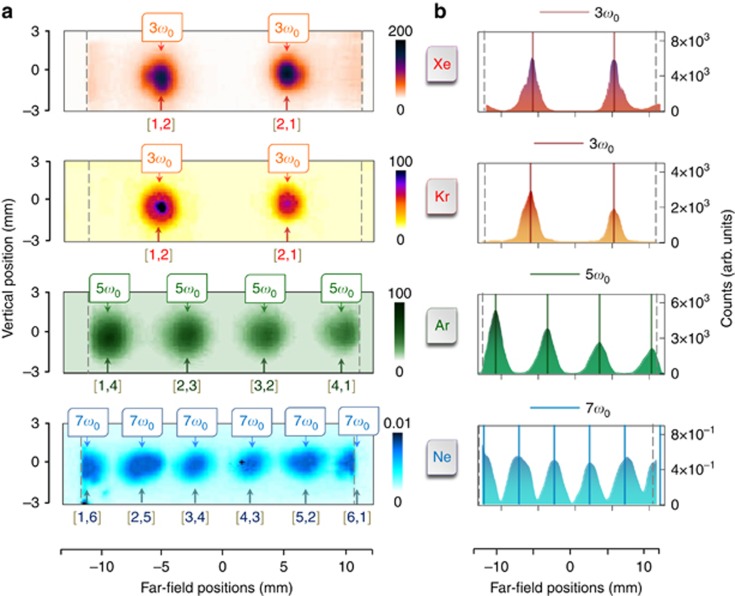
Non-collinear harmonic emission from rare gases. (**a**) Far-field emission patterns from Xe, Kr, Ar and Ne recorded under identical experimental conditions. The crossing angle *α* was chosen to be ~1.8^o^, and the pressure in the gas cell was maintained at 43 mbar. The relevant harmonics, their expected positions and the corresponding wave-mixing combinations [*m*,*n*] are indicated. (**b**) Vertically integrated line profiles (filled plots) of the far-field patterns indicate gas-specific harmonic emission. The dashed gray lines at the extremes indicate the beam block positions. The beam propagating towards the left was slightly more intense than the other, explaining the weak observed asymmetry. The photon flux of the most intense beamlet was determined to be 7 × 10^6^ photons per pulse for 3*ω*_0_ generated in Xe, 3 × 10^6^ photons per pulse for 3*ω*_0_ generated in Kr and 1 × 10^6^ photons per pulse for 5*ω*_0_ generated in Ar.

The photon flux, given in the caption of Figure 2, was determined as follows. The very low sensitivity of the MCP for the 400-nm fundamental beams makes it an effective transducer for the harmonics. The phosphor screen was simultaneously configured as an anode to measure the total generated charge. The number of photons per pulse *N*_*q*,*ν*_ contained in one beamlet of harmonic *q* is related to *N*_*q*,*e*_, the total number of generated electrons as *N*_*q*,*ν*_=*N*_*q*,*e*_/*Q*_*q*_, where *Q*_*q*_ is the detector quantum efficiency for photons of energy *qω*_0_, provided by the MCP manufacturer. *N*_*q*,*e*_ is related to the integrated fluorescence intensity *I*_*q*,*f*_ of the far-field profile on the phosphor screen as *I*_*q,f*_=*κN*_*q,e*_*G*_*V*_ through the MCP gain factor *G*_*V*_ (also provided by the manufacturer) at the bias voltage *V*. The constant *κ*, which includes the electron-to-photon conversion efficiency of the screen and image acquisition parameters is determined experimentally by selecting one beamlet with the help of physical masks in front of the detector and measuring the total number of electrons that reach the phosphor screen as 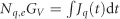, where *J*_*q*_(*t*) is the measured current. The integrated image intensity is thus related to the current as 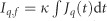 by the constant *κ*, enabling us to convert image intensities to photon numbers.

## Results and discussion

Figure 2 shows the far-field patterns of harmonic emission from two 400-nm beams using different rare gases as the target. The beams were apertured to a diameter of 3–4 mm using irises, corresponding to pulse energies of 70–90 μJ and peak intensities of 4–9 × 10^12^ W cm^−2^. The large Rayleigh range resulting from the focusing geometry (*f*/125 to *f*/160) corresponds to an extended longitudinal overlap with an effective medium length of *l*_m_=46 mm. The most striking feature in the far-field patterns is the emission of a single harmonic order, characteristic of the target gas. Xe and Kr emit only 3*ω*_0_, Ar distinctively generates 5*ω*_0_ beamlets and Ne dominantly generates 7*ω*_0_ emission. The suppression of above-threshold harmonics (5*ω*_0_ and 7*ω*_0_) in Xe and Kr can be readily understood as a consequence of their strong absorption above the ionization threshold caused by the extended propagation length in the semi-infinite gas cell. However, the absence of below-threshold orders (that is, 3*ω*_0_ in Ar, 3*ω*_0_ and 5*ω*_0_ in Ne) with their usual high propensities *S*_*q*_ at the single-atom level is unexpected at first sight.

**Figure 3:**
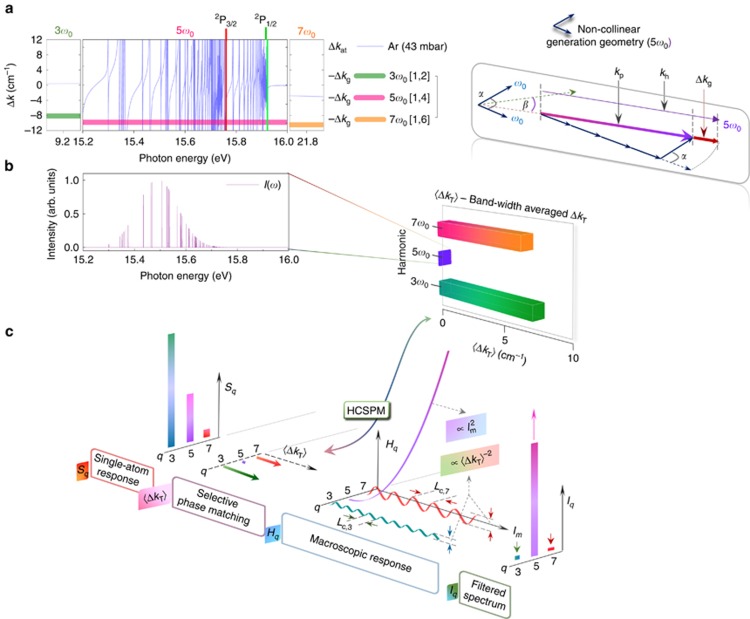
Spectral gating of NTH: the principle in Ar. (**a**) The geometric and atomic phase mismatches Δ*k*_g_ and Δ*k*_at_ for the case of 400-nm driving beams crossing at an angle *α*=1.8° in Ar at a pressure of 43 mbar are shown over the experimental bandwidth of the harmonics. For clarity, Δ*k*_g_ is considered only for the photon combinations [1,2], [1,4] and [1,6] corresponding to the harmonics 3*ω*_0_, 5*ω*_0_ and 7*ω*_0_, respectively. The finite numbers of perfectly phase-matched frequency regions correspond to the points of intersection of −Δ*k*_g_ (thick horizontal lines) with the atomic phase mismatch Δ*k*_at_ (blue line). The vector diagram on the right shows the origin of Δ*k*_g_ in non-collinear geometries towards the generation of 5*ω*_0_. (**b**) Calculated intensity spectrum of the macroscopic response which is dominated by frequency components corresponding to Δ*k*_T_=0. The horizontal bar diagram shows the intensity-weighted average 〈Δ*k*_T_〉 of the total phase mismatch Δ*k*_T_=Δ*k*_g_+Δ*k*_at_ over the bandwidth Δ*ω*_*q*_ of the respective harmonic orders. The near-zero value of 〈Δ*k*_T_〉 in the case of harmonic order 5 is attributed to the presence of intensity enhancements at zero crossings of Δ*k*_T_ within the Rydberg manifold. (**c**) Flow diagram illustrating the *in situ* spectral gating. The single-atom response *S*_*q*_ is subjected to High Contrast Selective Phase Matching (HCSPM) in macroscopic propagation, leading to a quadratic growth of 5*ω*_0_ over the length of the medium *l*_m_ as reflected by *H*_*q*_(*l*_m_). The non-phase-matched orders are scaled up in intensity for visibility. The resulting spectrum *I*_*q*_ at the end of the medium is monochromatized with high contrast.

To explain these surprising observations, we now consider in Figure 3 the phase-matching properties of NTH generated in a non-collinear geometry. We illustrate the case of Ar for which the harmonic order *q*=5 falls into the Rydberg manifold converging to the electronic ground state of Ar^+^. For ease of description, the analysis is restricted to the harmonic orders 3*ω*_0_, 5*ω*_0_ and 7*ω*_0_, although the principles are generally valid. The geometric phase mismatch Δ*k*_g_ for the harmonic order *q* between *k*_h_, (the *k*-vector for the harmonic wave) and *k*_p_ (the polarization wave resulting from the combined 400-nm driving beams with wave vector *k*_0_ crossing at an angle *α*) is given by Heyl *et al*^14^ (also see vector diagram in Figure 3a).


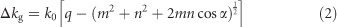


The values of −Δ*k*_g_ for *q*=3, 5 and 7 with *m*=1 are shown in Figure 3a (thick lines), along with the atomic phase mismatch Δ*k*_at_ (thin lines) for the experimental conditions (*α*=1.8° and gas cell pressure ~43 mbar) in Ar. The positive Gouy phase mismatch Δ*k*_G_≈0.05 cm^−1^ is smaller by more than an order of magnitude. Phase matching of a given harmonic order *q* is best discussed in terms of a single parameter, the intensity-weighted average of the total phase mismatch Δ*k*_T_=Δ*k*_g_+Δ*k*_at_ over the bandwidth Δ*ω*_*q*_, referred to as 〈Δ*k*_T_〉 and defined as:


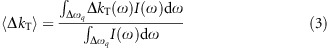


Here *I*(*ω*) is the intensity spectrum of the macroscopic response (see below for the evaluation procedure). For the illustrative case of Ar, 〈Δ*k*_T_〉 is shown in Figure 3b as a horizontal bar plot. The macroscopic intensity spectrum *I*(*ω*) (Figure 3b) displays pronounced maxima at the phase-matched frequencies within the Rydberg manifold for which Δ*k*_T_≈0. The intensity weighting on average therefore leads to 〈Δ*k*_T_〉≈0 for the 5*ω*_0_ band. We note that the calculated 〈Δ*k*_T_〉 significantly differs from a non-intensity-weighted average.

The averaging was performed over the spectral bandwidths measured in a collinear geometry (Supplementary Material; Supplementary Fig. S1). Ordinarily, the phase mismatch 〈Δ*k*_T_〉, varies little from one harmonic order to the next^20, 21, 22^. However, in the present case, 〈Δ*k*_T_〉, displays a narrow local minimum at 5*ω*_0_. The rapid variation of Δ*k*_at_ with photon energy, resulting from Rydberg resonances, leads to compensation of the large positive values of Δ*k*_g_, and therefore to phase-matched generation at these frequencies. The other orders retain high values of 〈Δ*k*_T_〉, because Δ*k*_g_ cannot be compensated by Δ*k*_at_. We refer to this phenomenon, prevailing in non-collinear generation geometries (see Supplementary Material for a comparison with collinear geometries), as ‘High-Contrast Selective Phase Matching’ (HCSPM), where phase-matched generation of a given harmonic is simultaneously accompanied by high phase mismatch of the adjacent ones. The observed gas-specific frequency selection of NTH (Figure 2) is a direct consequence of this phenomenon.

The emitted intensity *I*_*q*_ after propagation can be represented as a product of *S*_*q*_, the single-atom emission and a macroscopic response function *H*_*q*_(*l*_m_)^23^ as:


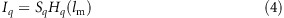


In the limit of very low values of the absorption cross-section *σ*_*q*_, applicable to NTH, *H*_*q*_(*l*_m_) has the following limiting functional forms^23^:


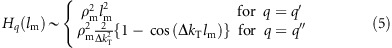


Here *q*′ corresponds to a phase-matched harmonic order, *q*″ is a non-phase-matched order and *ρ*_m_ is the medium density. Whereas the intensity of the phase-matched order grows quadratically within the medium, the intensity of the adjacent harmonics oscillates between a maximum value of 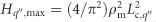 and zero (Figure 3c). Here 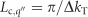 is the coherence length of the non-phase-matched orders *q*″. Note that *H*_*q*″_,max is inversely proportional to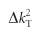, providing an independent and sensitive handle for determining the maximum intensity growth within the medium for *q*″. The large values of 〈Δ*k*_T_〉 for the non-phase-matched orders suppress their intensities and hence allow for frequency gating of harmonic emission to a single order at the end of the medium.

Figure 3c summarizes these concepts for the case of argon. The single-atom intensity distribution *S*_*q*_ is subjected to HCSPM and the macroscopic response function *H*_*q*_(*l*_m_) restricts the beamlets in the emission cone to solely the phase-matched order 5*ω*_0_ in the final spectrum *I*_*q*_. Applying the same principle to neon explains the selective generation of 7*ω*_0_ and the suppression of 3*ω*_0_ and 5*ω*_0_, leading to the emission of six synchronized beams with a photon energy of 21.7 eV, as observed. This technique relies on (a) enhancing 〈Δ*k*_T_〉 for the non-NTH orders and (b) large propagation lengths to translate HCSPM to a high degree of spectral contrast in *I*_*q*_. While in the non-collinear geometry, the use of 400-nm light fields with enhanced *k*_0_ (Equation (2)) and the overlap of *qω*_0_ with the Rydberg manifold fulfills condition (a), a large wave-mixing length *l*_m_ (possible in semi-infinite gas cell) with small crossing angle *α* was crucial towards condition (b) and observing this mechanism in our experiments. A comparison of spectral gating in collinear and non-collinear geometries is shown in the Supplementary Material (Supplementary Fig. S2).

**Figure 4:**
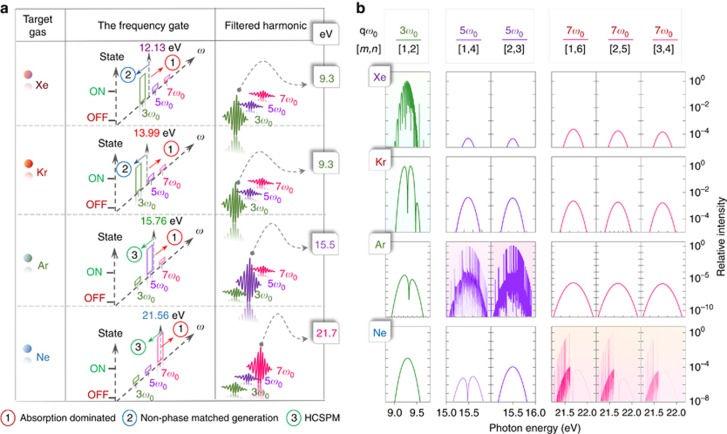
NTH frequency gating and tunability in rare gases. (**a**) The spectral gating (rectangles in column 2) in non-collinear emission can be tuned by changing the generation gas leading to selection of 9.3 eV (in Xe and Kr), 15.5 eV (in Ar) and 21.7 eV (in Ne) photons. A rectangle with large height (‘on’ state) represents the presence and shorter rectangles (‘off’ state) the absence of a specified harmonic order in the non-collinear emission. The vertical arrow for each target gas corresponds to the respective ionization limit. Further, the circled numbers highlight the dominant mechanisms in each spectral zone (diagonal blue, red and green arrows) for the respective gas. Column 3 pictorially shows the spectral content of the harmonic emission. (**b**) Predicted spectral intensities *I_q_*(*ω*) for rare gases for three different orders *q* and their respective wave-mixing combinations [*m*,*n*].

To validate these principles further, we have performed calculations to evaluate the spectral composition of the non-collinear beamlets after propagation and show the results in Figure 4. These calculations take into account all phase-matching characteristics as well as absorption by the medium in calculating the harmonic intensities *I*_*q*_(*ω*). Arriving at *I*_*q*_(*ω*) involved the experimental determination of *S*_*q*_(*ω*) and the calculation of *H*_*q*_(*ω*).

The atomic response *S*_*q*_ was directly measured in complementary experiments using a collinear geometry (Supplementary Material; Supplementary Fig. S1) and was represented by:


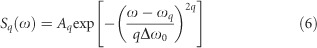


Here Δ*ω*_0_ is the full-width-at-half-maximum bandwidth of the spectrum of the fundamental driving pulse. The pre-factors *A*_*q*_ were obtained from an experimental determination of the integrated single-atom response 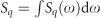 (Supplementary Material). The atomic contribution towards the macroscopic response function 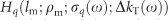 depends on the atomic phase mismatch 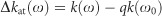 and absorption *σ*(*ω*), where *k*(*ω*) and *σ*(*ω*) are the dispersion relationship and the frequency-dependent absorption in the medium, respectively. *k*(*ω*) and *σ*(*ω*) are related to the real and imaginary parts, respectively, of the complex refractive index *n*(*ω*)=*n*_R_(*ω*)+i *n*_I_*(ω*) as follows:


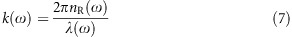


and


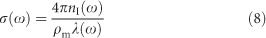


Here and in what follows below, *ρ*_m_ is the atomic density and *ω*_th_ is the energy corresponding to the ionization threshold in atomic units. Both the real and imaginary parts of the refractive index have contributions from the continuum and from atomic resonances located at photon energies *ω*_*j*_. The resonance contributions are calculated within the framework of the Lorentz oscillator model for polarization using the following coupled equations:


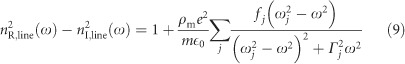


and


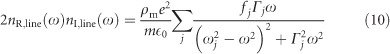


The continuum contributions are given by


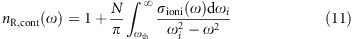



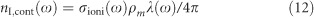


Here *f*_*j*_ is the oscillator strength for the *j^th^* dipole-allowed transition of photon energy *ω*_*j*_. Further, *Γ*_*j*_=1/*τ*_*j*_ is the natural linewidth of the transition, *e* and *m* are the charge and mass of electron, *∈*_0_ is the permittivity of vacuum, *σ*_ioni_ (*ω*) is the single-photon ionization cross-section at photon energy *ω*.

In the calculations, we used a generation length equal to the longitudinal beam overlap of 46 mm, an additional effective length for absorption of 10 mm, a crossing angle *α*=1.8° and a pressure of 43 mbar. The spectral evaluation of intensities *I*(*ω*) near the threshold was performed over a grid with a step size of 25 μeV small enough to resolve resonances. The predicted intensities *I_q_*(*ω*) are shown in Figure 4b.

Overall, the calculated intensities indeed predict the generation of a single dominant order, specific to the chosen medium. The enhancement of 5*ω*_0_ in Ar and 7*ω*_0_ in Ne is caused by their overlap with Rydberg manifolds converging to their lowest ionization thresholds and leads to the formation of resonance-enhanced structures observed in collinear 400-nm harmonic generation (Supplementary Material; Supplementary Fig. S1). The proximity of 3*ω*_0_ to the position of the 6-s resonance of Xe (9.56973 eV) causes a rapid variation of the phase mismatch Δ*k*_T_ across the bandwidth of the harmonic, high enough to modulate the spectrum. In Kr, the harmonic 3*ω*_0_ is further away from the closest 5-s resonance (10.0324 eV) and hence has a lower variation in Δ*k*_T_ that leads to a weaker modulation of the spectrum. In both cases, however, the strong absorption just above the ionization threshold plays an assisting role by additionally suppressing the intensities of above-threshold orders.

Finally, we studied the duration of the generated VUV/XUV pulses. We measured the duration of 3*ω*_0_ pulses generated in Xe and 5*ω*_0_ pulses generated in Ar in a collinear geometry to be ~78  fs (Supplementary Material; Supplementary Fig. S4). Measurements performed in a non-collinear geometry for 5*ω*_0_ pulses generated in Ar lead to a consistent result (Supplementary Material; Supplementary Fig. S3). The spectrally-resolved far-field profile from Ar is shown in the Supplementary Material (Supplementary Fig. S5). These results show that the presence of resonances does not prevent the emission of ultrashort VUV/XUV laser pulses that will therefore find applications in time-resolved photoemission experiments.

## Conclusions

In summary, we devised a new scheme for all-optical frequency gating and simultaneous beam splitting of VUV/XUV radiation inside the generation medium. Our scheme exploits the phase-matched generation facilitated by resonances close to the ionization limit and the concomitant phase mismatch of adjacent harmonics in a non-collinear geometry. The scheme of HCSPM introduced in this article can transform the monotonically decreasing single-atom response *S*_*q*_ to a spectrally filtered function *I*_*q*_ as a consequence of the non-collinear macroscopic response function *H*_*q*_. We demonstrated both experimentally and theoretically that the effective realization of HCSPM requires a non-collinear geometry. The high contrast of the filter relies on the long medium length *l*_m_ made possible in a semi-infinite gas cell. The scheme of HCSPM reported here is generally applicable and can be used to spectrally filter a range of photon energies within the Rydberg manifolds. This scheme could become an alternative to the conventional dispersion-based grating monochromators^24^ or multilayer mirrors^25^ due to its all-optical nature and simple layout. The non-collinear filter has the added advantage of naturally separating the harmonic radiation from the fundamental and the availability of multiple synchronized beamlets for multi-pulse pump-probe schemes^26, 27^. Consequently, this scheme may enable new approaches to frequency-selective outcoupling from femtosecond enhancement cavities^28, 29, 30^, paving the way to MHz repetition-rate spectrally pure XUV sources with possible applications in ultrafast time-resolved spectroscopy, XUV holography and microscopy.
