# Supplementary material for: In situ frequency gating and beam splitting of vacuum- and extreme-ultraviolet pulses
Source: Light Sci Appl. 2016 Nov 18;5(11):e16170–. doi: 10.1038/lsa.2016.170 (PMC6059825; doi:10.1038/lsa.2016.170)
Supplement: Supplementary Information [file lsa2016170x1.pdf]

Supplementary Material for
In situ frequency gating and beam splitting of vacuum- and extreme-ultraviolet pulses

Rajendran Rajeev, Johannes Hellwagner, Anne Schumacher, Inga Jordan, Martin Huppert, Andres Tehlar, Bhargava Ram Niraghatam, Denitsa Baykusheva, Nan Lin, Aaron von Conta and Hans Jakob Wörner*

Laboratory of Physical Chemistry, Vladimir-Prelog-Weg 2, ETH Zürich, 8093 Zürich Switzerland

(Dated: June 21, 2016)

* hwoerner@ethz.ch

CONTENTS

A. Collinear high-harmonic generation with 400-nm fields: observation of resonance-enhanced structures 2
B. Frequency gating: comparison of collinear and non-collinear geometries 4
C. Pulse-duration measurements 5
D. Tuning the non-collinear angle for contrast enhancement: the case of neon 9

A. Collinear high-harmonic generation with 400-nm fields: observation of resonance-enhanced structures

The spectral gating described in the main text partially relies on the possibility of phase-matched harmonic generation in the vicinity of resonances. The use of a 400 nm driver leads to resonance enhancement in Ar and Ne because the bandwidths of the $5\omega_0$ and $7\omega_0$ emissions encompass the corresponding Rydberg manifolds converging to their lowest ionization thresholds (see Figure S1). We experimentally demonstrate this fact by measuring the harmonic spectra in a collinear geometry. The experiment involved insertion of a reflective grating in the line-of-sight of the collinear beam in order to disperse the harmonics onto the MCP detector. The results of these experiments are shown in Figure S1. The images in Figure S1 **a-d** represent the spectrally-dispersed harmonics as observed on the MCP. The spectra corrected by the Jacobian accounting for conversion from wavelength to photon energy are shown at the bottom. The boxes above the spectra show the dipole-allowed transitions in the respective gases, along with the spectral position of the expected harmonic emission from the 400 nm driver. These measurements show the expected resonance-enhanced features in $5\omega_0$ (Ar) and $7\omega_0$ (Ne). The emergence of these resonances in the proximity of the ionization thresholds of the two gases is a consequence of phase matching ($\langle \Delta k_T \rangle \approx 0$). The large range of negative values of Δk_{at} within the Rydberg manifold offer multiple spectral regions for compensating the positive geometric phase mismatch. In contrast, the harmonics emitted from Xe and Kr invariably lie in spectral regions characterized by positive Δk_{at}, preventing the formation of resonance-enhanced structures.

The spectral distribution observed in the collinear geometry, I_q^{col}, and shown in Figure S1 is used as an input to calculate the final spectrum I_q from the non-collinear geometry, shown

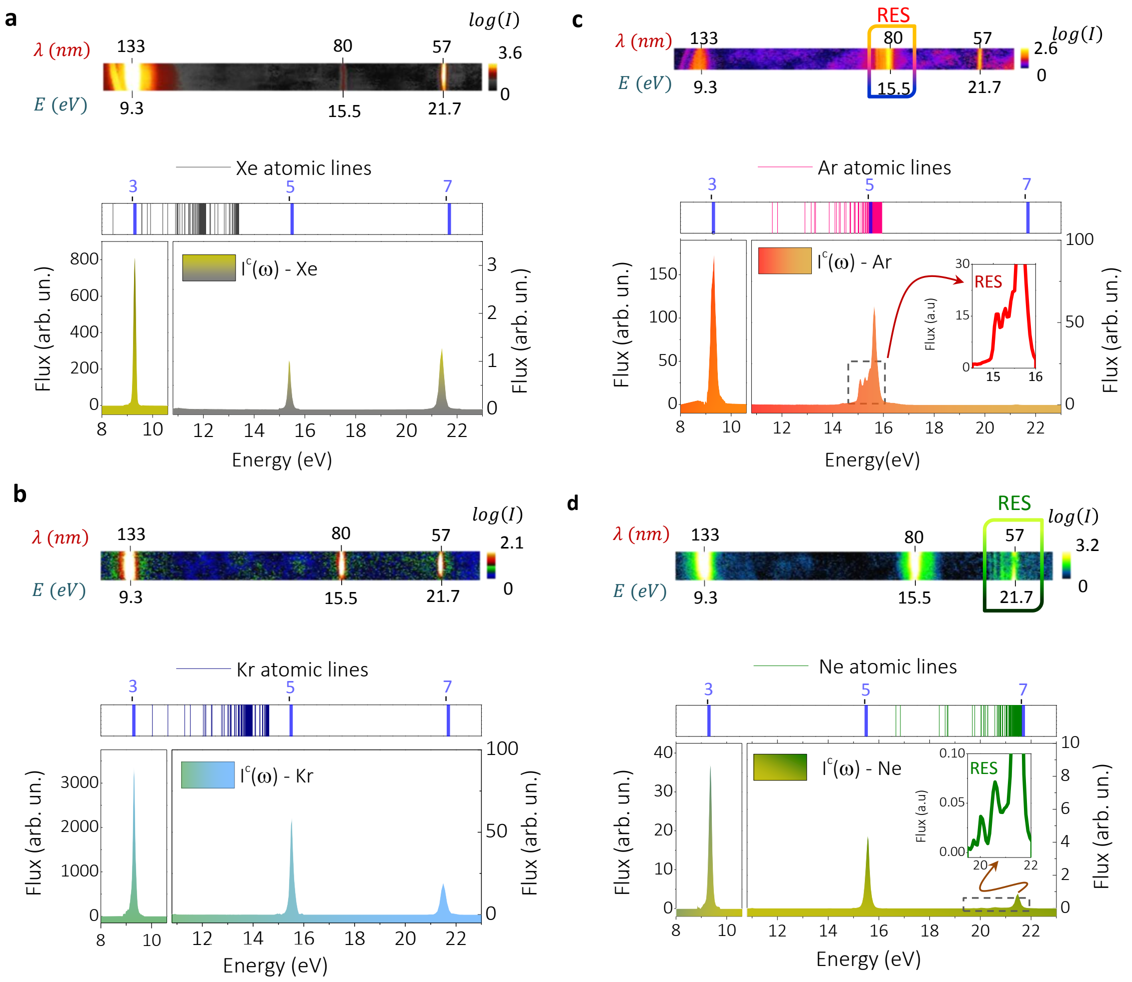

Figure S1: **Harmonic spectra in collinear geometry emitted from noble gases using 400 nm driving fields: (a)** Xe, **(b)** Kr, **(c)** Ar, **(d)** Ne. The image in each figure corresponds to the raw image on the MCP after dispersion using a reflective grating. The second inset displays the one-photon-allowed resonances in the respective gas and the expected harmonic emission using a 400-nm driving field (in light blue). In **(c)** and **(d)** the emergence of resonance-enhance structures (RES) around $5\omega_0$ and $7\omega_0$ (see insets) close to the Rydberg manifold of Ar and Ne respectively is clear.

in Figure 4 of the main text. The observed collinear spectrum I_q^{col} depends on S_q, the single-atom response, and the macroscopic collinear response $H_q^{col}(l_m)$, over the medium length l_m, and is given by

$$I_q^{col} = S_q H_q^{col}(l_m). \tag{S1}$$

We thus determine S_q from an experimental measurement of I_q^{col} and the calculated value of $H_q^{col}(l_m)$.

B. Frequency gating: comparison of collinear and non-collinear geometries

The efficiency of the spectral gating demonstrated in the main text partially relies on the characteristics of the non-collinear geometry. The slow monotonic intensity decrease with increasing harmonic order shown in Figure S1 excludes frequency gating in a collinear geometry. Here, we make a comparative analysis of non-collinear with collinear generation geometries to establish this fact. Figure S2 summarizes the results of these calculations. To simplify the comparison of collinear and non-collinear generation geometries, we restrict the discussion to non-collinear generation pairs [1,2],[1,4], and [1,6] corresponding to harmonic orders 3, 5 and 7 respectively. The non-collinear calculations assume a crossing angle $\alpha = 1.8^\circ$ as in the experiment. Figure S2a shows the total phase mismatch across the three harmonic bandwidths corresponding to $3\omega_0$, $5\omega_0$ and $7\omega_0$ for the collinear (top) and the non-collinear (bottom) cases. Clearly in both cases, within the Rydberg manifold around the $5\omega_0$ emission, there are multiple zero crossings, allowing phase-matched generation. However, the particularity of the non-collinear geometry is in the enhancement of the phase mismatch for the adjacent harmonics, indicated by the arrows in Figure S2a. The phase mismatch $\langle \Delta k_T \rangle$, obtained by averaging over the experimental spectral bandwidths from Figure S1a, is shown in Figure S2b. For the below-threshold harmonic $3\omega_0$, the non-collinear geometry leads to a $\langle \Delta k_T \rangle$ which is 20 times larger than in the collinear geometry. Further, the large contrast in $\langle \Delta k_T \rangle$ between the NTH and non-NTH orders in the non-collinear geometry translates to a selective growth of one order, simultaneously with the suppression of the adjacent harmonics, as indicated in Figure S2c. The suppression of the non-enhanced orders is comparatively much lower in the case of collinear geometries. This is the reason for the observation of such orders in the collinear spectrum in Figure S1 and the associated absence of spectral gating as shown in Figure S2d.

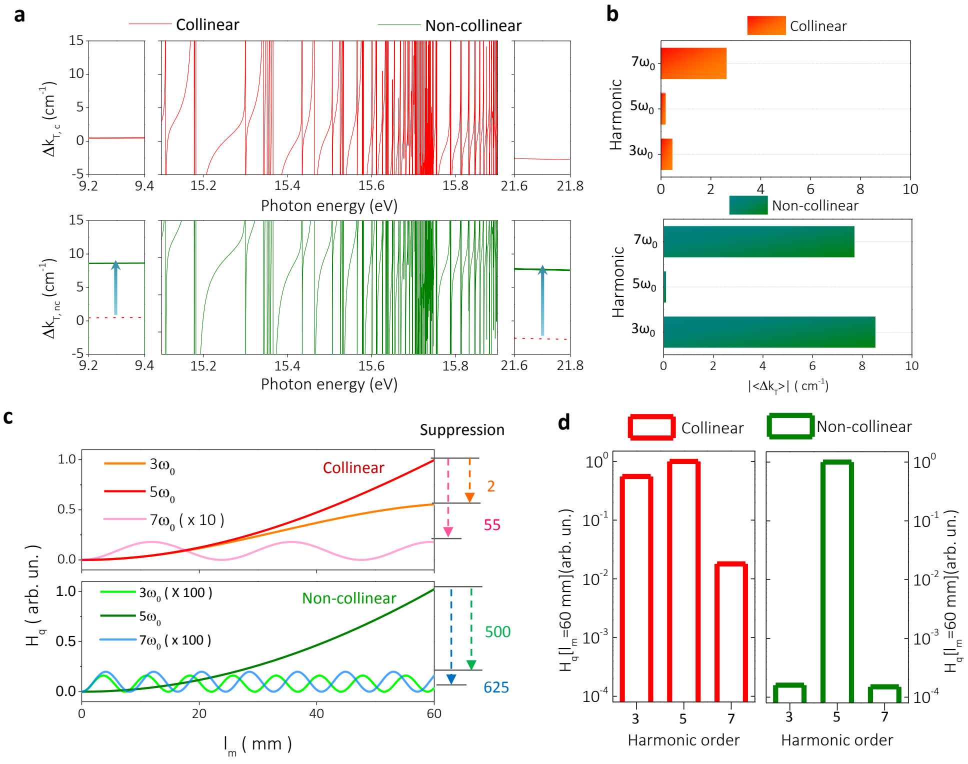

Figure S2: **Spectral gating in collinear and non-collinear geometries - the case of Ar. (a)** The total phase mismatch Δk_T for collinear and non-collinear geometries for photon energies corresponding to harmonic orders $3\omega_0$, $5\omega_0$ and $7\omega_0$. The blue arrow indicates the enhancement in Δk_T offered in a non-collinear geometry over the collinear one. **(b)** Magnitude of the bandwidth-averaged $\langle \Delta k_T \rangle$ for collinear and non-collinear cases. **(c)** The response functions $H_q(l_m)$ in collinear (top) and non-collinear (bottom) schemes showing the growth of the harmonic orders as a function of medium length l_m. **(d)** Macroscopic response function H_q for both geometries showing that frequency gating is effective only in the non-collinear case.

C. Pulse-duration measurements

The presence of resonance-enhanced structures in the high-harmonic spectra naturally raises the question of the duration of the corresponding VUV/XUV pulses. We have addressed this question by performing two complementary types of cross-correlation measure-

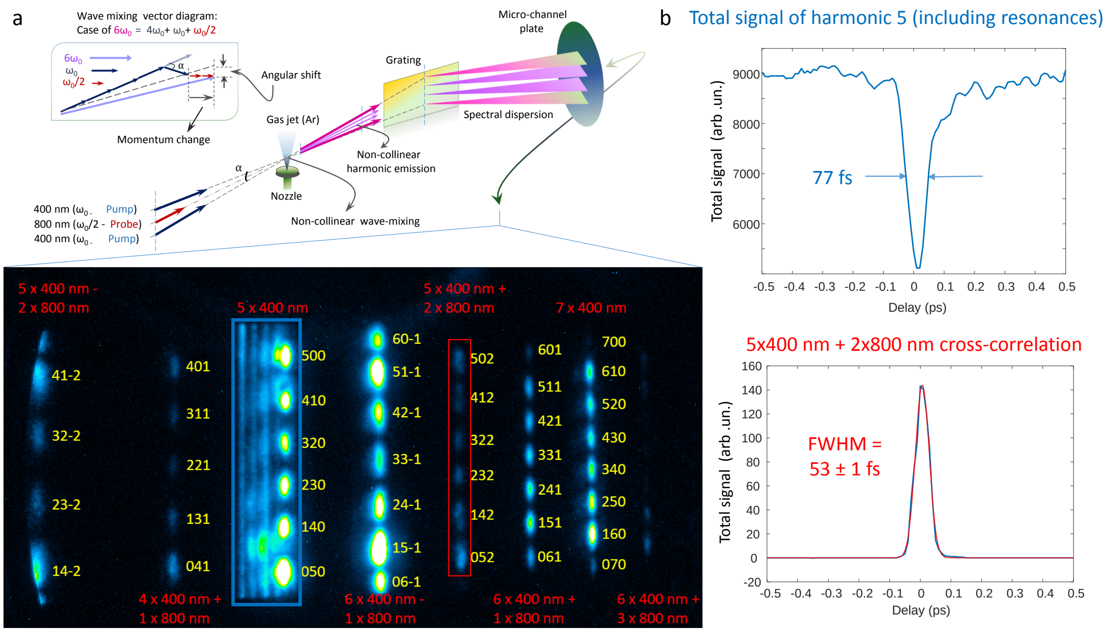

Figure S3: **Cross-correlation measurement of the XUV generation process (a)**, Experimental setup used for an "in-situ" measurement of the XUV generation process in a thin gas jet and net photon numbers ($n_1 n_2 n_3$) contributed by the 400(top)/400(bottom)/800 nm beams, **(b)**, cross-correlation signals obtained by integrating all signals related to $5\omega_0$ (blue box in Figure S3a, top panel in Figure S3b) or $6\omega_0$ (red box in Figure S3a, bottom panel in Figure S3b).

ments.

In the first experiment, we have realized an all-optical "in-situ" measurement of the XUV generation process. We have combined non-collinear generation in a thin gas jet (~ 0.5 mm) with a grating spectrometer and have added a perturbative (intensity $\sim 10^{11}$ W cm^{-2}) 800-nm, 30 fs pulse, bisecting the angle formed by the two 400-nm beams (see Figure S3a). We have varied the delay between the 800-nm and the two synchronized 400-nm pulses and have recorded the wave-mixing signals resulting from the temporal overlap of all 3 beams. The spatially and spectrally resolved pattern is shown in Figure S3a. Each of the spots on the detector is assigned in terms of three integers (n_1 n_2 n_3), representing the net number n_1 of 400-nm photons from the lower beam, the number n_2 of 400-nm photons from the upper beam and the number n_3 of 800-nm photons, respectively, contributing to the signal. Figure S3b shows the observed cross correlation signals, obtained by integrating all signals of $5\omega_0$, including the resonance lines (blue box in Figure S3a) or all signals associated with

wave-mixing of 5 × 400 nm + 2 × 800 nm photons (red box in Figure S3a). The 800-nm pulse is found to perturb the emission of the XUV pulses over a duration of ∼77 fs and to yield additional frequencies, e.g. at the position $6\omega_0$, over a duration of 53 fs. This measurement shows that the emission of the XUV pulses is temporally confined to less than ∼77 fs, although a weak asymmetric tail is observed to positive delays, corresponding to the IR pulse arriving after the UV pulse. If the emission was extending over a longer period, the IR pulse could still perturb the generation process which would manifest itself as both a longer-lasting depletion of $5\omega_0$ and a longer-lasting emission of the wave-mixing signals. The shorter duration of the cross correlation measured in the wave-mixing signal is attributed to the non-linearity of the two-photon interaction with the IR pulse.

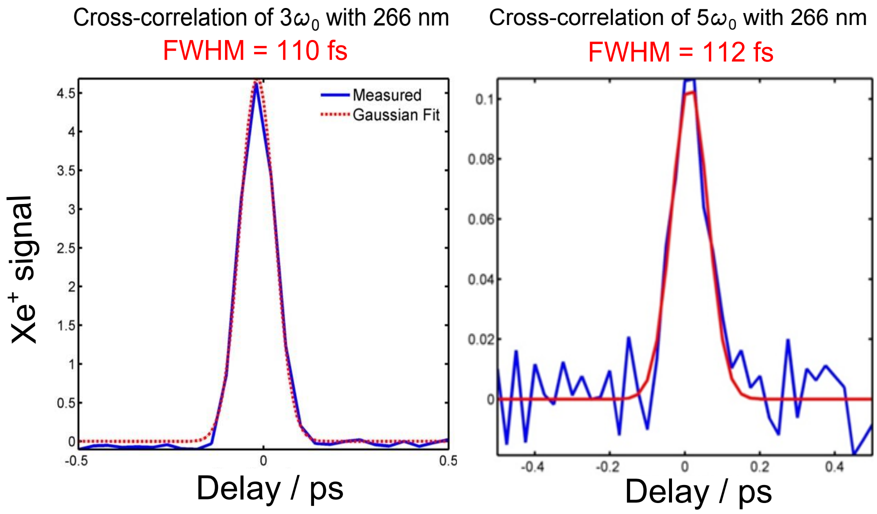

Figure S4: Cross-correlation between $3\omega_0$ generated in Xe or $5\omega_0$ generated in Ar with an 80-fs pulse centered at 266 nm, through $1+1'$ non-resonant photoionization of Xe atoms in a supersonic expansion.

In the second experiment, we have measured the duration of the $3\omega_0$ pulse generated in xenon and the $5\omega_0$ pulse generated in argon. Both measurements were performed using the semi-infinite gas cell described in the main text, in an "ex-situ" experiment through non-resonant 1+1' two-color photoionization of xenon atoms in a supersonic expansion. These cross-correlation measurements were performed using an 80-fs pulse centered at 266 nm. We have done these measurements in a collinear generation geometry, under the same conditions as Figure S1. This measurement addresses the question whether resonance-enhanced structures affect the duration of the generated VUV/XUV pulses. The results are show in

Figure S4. The observed cross-correlations amount to 110 fs in the case of $3\omega_0$ generated in xenon and 112 fs in the case of $5\omega_0$ generated in argon. Taking into account the 80-fs duration of the 266-nm pulse, we obtain a duration of 75 fs for $3\omega_0$ generated in Xe and 78 fs for $5\omega_0$ generated in Ar. This result is consistent with the "in-situ" measurements described above.

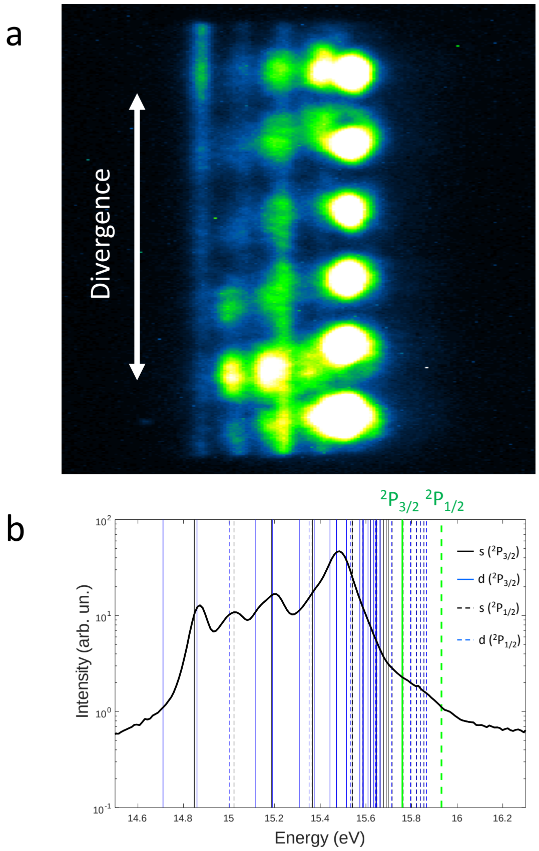

Figure S5: **(a)** Frequency-resolved far-field profile of $5\omega_0$ generated in argon, obtained by dispersing the emission from two crossed 400-nm beams with a flat-field grating spectrometer. **(b)** Integral over the spatial divergence of Figure S5a, displaying the spectrum of $5\omega_0$. The two ionization thresholds corresponding to Ar^+ in the $^2P_{3/2}$ and $^2P_{1/2}$ state are indicated, together will all s and d Rydberg states with $J = 1$ converging to these two thresholds.

These two sets of measurements show that high-harmonic generation in the presence of resonances still leads to the emission of sub-80 fs pulses. Although our phase-matching calculations (Figure 3 and 4 of the main text) suggest the appearance of sharp structures in the macroscopic response, such sharp features were not observed in our measurements (Figures S1 and S3). Figure S5 shows a magnification of the far-field profile of the region

of Figure S3 corresponding to $5\omega_0$ emission. We observe resonance-enhanced structures in both $5\omega_0$ (Ar) and $7\omega_0$ (Ne, not shown) that are closely related to the structures observed in the collinear spectra. The positions, widths and intensities of these structures are very similar to the collinear case. Our measurements show that the presence of resonances does not prevent the emission of ultrashort VUV/XUV laser pulses that will find applications in time-resolved photoemission experiments.

D. Tuning the non-collinear angle for contrast enhancement: the case of neon

The final spectrum I_q is a product of the macroscopic response function H_q with the single-atom response S_q. Non-collinear generation at resonant frequencies creates a spectral gate for H_q. However, this does not necessarily translate to spectral filtering in Ne, owing to the fact that the single-atom response S_q at $7\omega_0$ (21.7 eV) is substantially weaker than that at $3\omega_0$ (9.3 eV). We performed calculations of the final spectrum generated in Ne to verify this. We chose to parametrize the final spectrum $I_q(\alpha)$ in terms of the non-collinear crossing angle α. The results of these calculations are shown in Figure S6a. The operating angle in the experiments was $\alpha_{exp} \sim 1.8°$. Around this angle, I_3(maximum value of the oscillating intensity) and I_7 are within the same order of magnitude, while I_5 is highly suppressed (for visibility I_5 is scaled up by a factor of 10). While the maxima in I_3 reach values close to I_7, they have a different functional dependence on α. The intensity $I_7(\alpha)$ is dominated by perfectly phase-matched contributions and therefore monotonically decreases with α, owing to decreasing beam overlap and hence effective medium length. In contrast, $I_3(\alpha)$ has an oscillatory behavior because none of its frequency components are phase-matched. This oscillatory behavior can be exploited to suppress $3\omega_0$ by fine-tuning the angle α. The maximal suppression of I_3 happens at $\alpha = 1.79°$, as shown in the inset of Figure S6a.

We confirm this prediction experimentally by capturing the non-collinear emission in Ne at two different crossing angles differing by $\Delta\alpha \sim 0.04°$. This small change in the angle was achieved by modifying the inter-beam separation by $\sim 250\ \mu$m using the high precision stage 2 shown in Figure 1 of the main text. The results of these experiments are shown in Figure S6b. The emission from Ne for a certain crossing angle $\alpha_{exp} \sim 1.8°$, comprises of $3\omega_0$ in addition to the expected $7\omega_0$ (top image in Figure S6b). However, by tuning the crossing angle to $\alpha_{exp} - \Delta\alpha$ the far-field emission pattern changes, with suppression

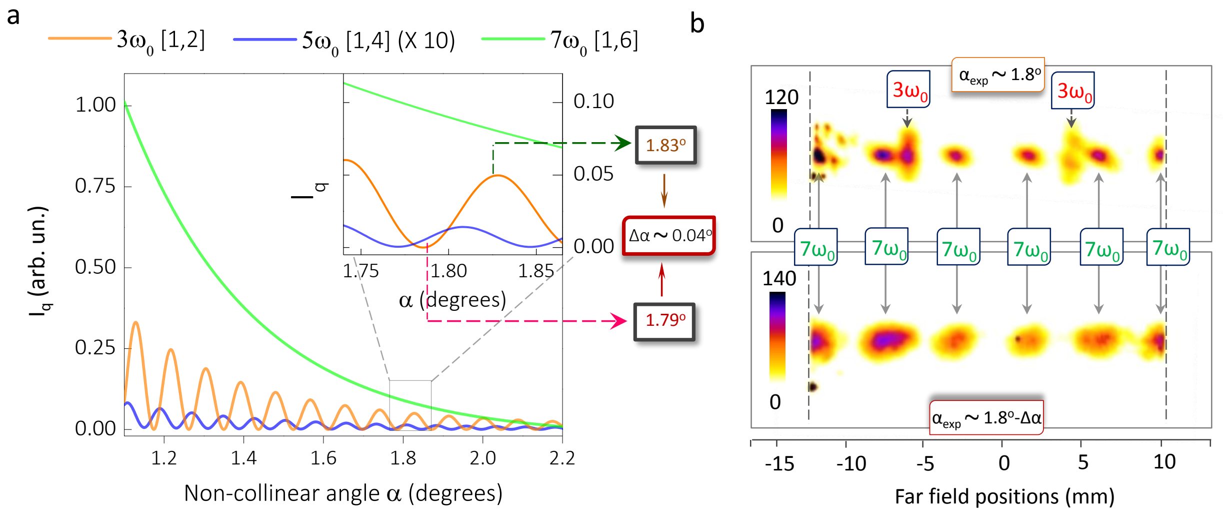

Figure S6: **Frequency gating in Ne: Angular tuning (a)** Calculated intensities of the harmonics $I_q(\alpha)$ as a function of α in Ne. The inset shows the variation at the operating angle around 1.8°. While I_5 is highly suppressed with respect to I_7, I_3 assumes values comparable to I_7 in spite of frequency gating of NTH. This is attributed to the large value of S_3 in comparison with S_7 in Ne. This can be suppressed by tuning the angle, as I_3 being non-phase-matched, oscillates and hence allows detuning in intensity as seen at $\alpha = 1.79°$. **(b)** The conical emission in Ne at two crossing angles showing the possibility of $3\omega_0$ suppression by tuning the experimental crossing angle α_{exp} by an amount $\Delta\alpha$ predicted in calculations. The image above shows the presence of $3\omega_0$ in addition to the expected $7\omega_0$ at $\alpha_{exp} \sim 1.8°$. The image below shows suppression of $3\omega_0$ emission when the angle is lowered by $\sim 0.04°$, allowing the emission of only $7\omega_0$ in Ne.

of the $3\omega_0$ beamlets as predicted by the calculations. This leads to a dominant emission of $7\omega_0$ within the cone (bottom image in Figure 3Sb). The experimental results for Ne shown in Figure 2 of the main text were obtained through this angular tuning. The control of harmonic emission by angular tuning in Ne extends the possibilities of spectral gating. This opportunity is particularly important to frequency gate higher order harmonics, where frequency isolation amounts to suppression of multiple lower-order harmonics. On the same note, selection of the $5\omega_0$ order in Ar did not require angular tuning as only the close-lying $3\omega_0$ harmonic needed to be suppressed, for which the disparity between S_3 and S_5 (the NTH order) is much lower than between S_3 and S_7 in Ne. In fact, it was observed experimentally

that $5\omega_0$ was cleanly selected at all angles α_{exp}.
